# The World Heart Federation Global Study on COVID-19 and Cardiovascular Disease

**DOI:** 10.5334/gh.950

**Published:** 2021-04-19

**Authors:** Karen Sliwa, Kavita Singh, Lana Raspail, Dike Ojji, Carolyn S.P. Lam, Friedrich Thienemann, Junbo Ge, Amitava Banerjee, L. Kristin Newby, Antonio Luiz P. Ribeiro, Samuel Gidding, Fausto Pinto, Pablo Perel, Dorairaj Prabhakaran

**Affiliations:** 1 Division of Cardiology, Department of Medicine, Faculty of Health Sciences, Groote Schuur Hospital, Cape Town, ZA; 2 Hatter Institute for Cardiovascular Research in Africa & CHI, Faculty of Health Sciences, University of Cape Town, South Africa, World Heart Federation, ZA; 3 Public Health Foundation of India, Gurugram, Haryana, IN; 4 World Heart Federation, CH; 5 Department of Medicine, Faculty of Clinical Sciences, University of Abuja, and University of Abuja Teaching Hospital, NG; 6 National Heart Center Singapore and Duke-National University of Singapore, SG; 7 Department of Cardiology, University Medical Center Groningen, University of Groningen, Groningen, NL; 8 Hatter Institute for Cardiovascular Research in Africa and Department of Medicine, Faculty of Health Sciences, University of Cape Town, ZA; 9 Department of Internal Medicine, University Hospital Zurich, University of Zurich, CH; 10 Department of Cardiology, Zhongshan Hospital, Fudan University. Shanghai Institute of Cardiovascular Diseases, Shanghai, CN; 11 University College London, UK; 12 Duke Clinical Research Institute, Durham, US; 13 Cardiology Service and Telehealth Center, Hospital das Clínicas, and Department of Internal Medicine, Faculdade de Medicina, Universidade Federal de Minas Gerais, Belo Horizonte, BR; 14 Santa Maria University Hospital, CAML, CCUL, Faculdade de Medicina da Universidade de Lisboa, Lisbon, PT; 15 Department of Non-communicable Disease Epidemiology, London School of Hygiene & Tropical Medicine, World Heart Federation, UK; 16 Centre for Control of Chronic Conditions, Public Health Foundation India, World Heart Federation, London School of Hygiene & Tropical Medicine, UK

**Keywords:** COVID-19, registry, cohort, survey, coronavirus, cardiovascular disease, rheumatic heart disease, chagas disease, HIV

## Abstract

**Background:**

The emergence of novel coronavirus disease 2019 (COVID-19), caused by the Severe Acute Respiratory Syndrome-Coronavirus-2 (SARS-CoV-2), has presented an unprecedented global challenge for the healthcare community. The ability of SARS-CoV-2 to get transmitted during the asymptomatic phase, and its high infectivity have led to the rapid transmission of COVID-19 beyond geographic regions facilitated by international travel, leading to a pandemic. To guide effective control and interventions, primary data is required urgently, globally, including from low- and middle-income countries where documentation of cardiovascular manifestations and risk factors in people hospitalized with COVID-19 is limited.

**Objectives:**

This study aims to describe the cardiovascular manifestations and cardiovascular risk factors in patients hospitalized with COVID-19.

**Methods:**

We propose to conduct an observational cohort study involving 5000 patients recruited from hospitals in low-, middle- and high-income countries. Eligible adult COVID-19 patients will be recruited from the participating hospitals and followed-up until 30 days post admission. The outcomes will be reported at discharge and includes the need of ICU admission, need of ventilator, death (with cause), major adverse cardiovascular events, neurological outcomes, acute renal failure, and pulmonary outcomes.

**Conclusion:**

Given the enormous burden posed by COVID-19 and the associated severe prognostic implication of CVD involvement, this study will provide useful insights on the risk factors for severe disease, clinical presentation, and outcomes of various cardiovascular manifestations in COVID-19 patients particularly from low and middle income countries from where the data remain scant.

## Introduction

**Figure 1:**
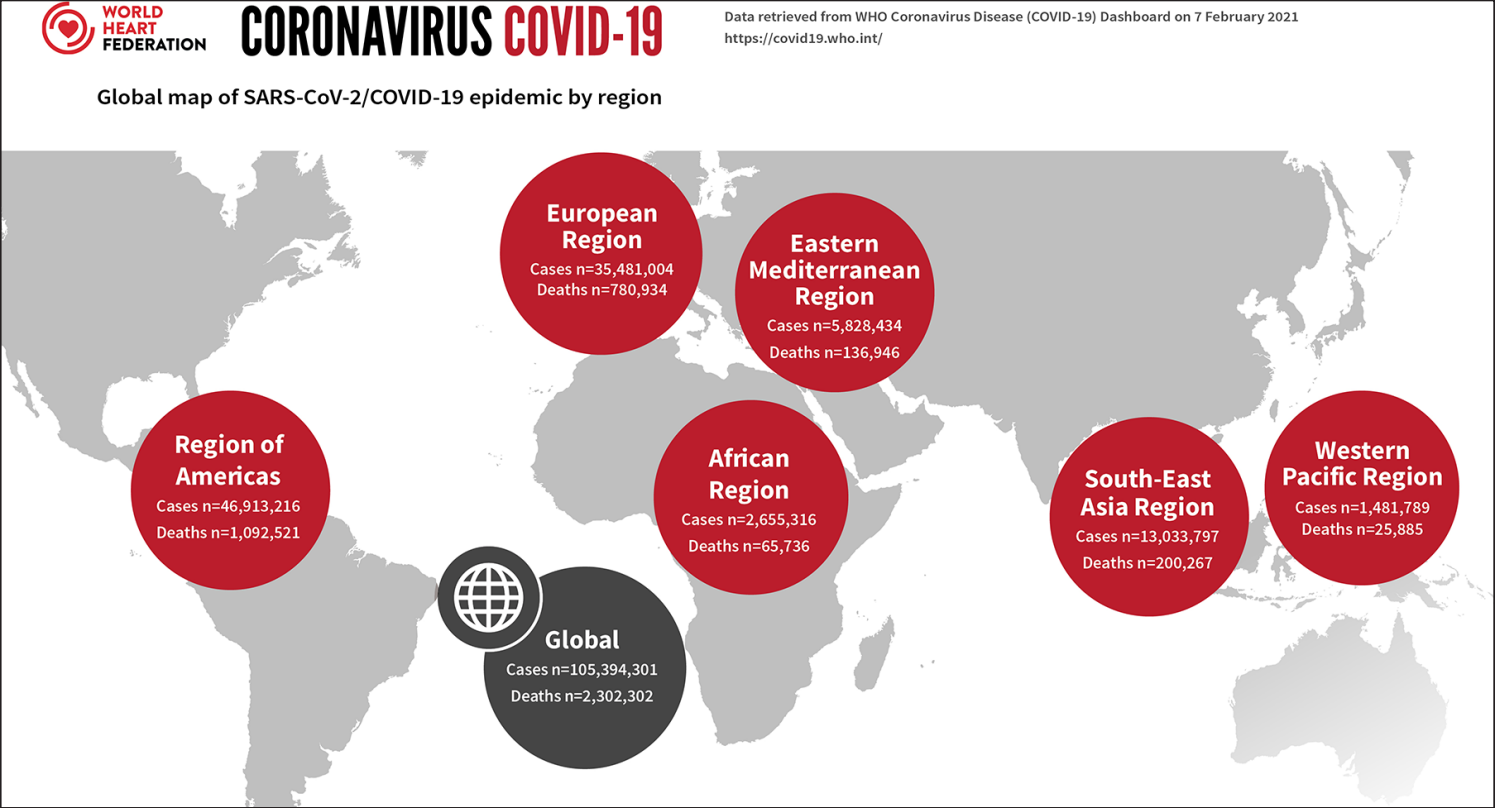
Global Map of COVID-19 pandemic by region; adapted *from World Heart Federation Briefing on Prevention: Coronavirus Disease 2019 (COVID-19) in Low-Income Countries* [4] and *Management of Cardiovascular Disease Patients With Confirmed or Suspected COVID-19 in Limited Resource Settings* [5].

The coronavirus disease 2019 (COVID-19), was declared a pandemic by the World Health Organization (WHO) on March 11^th^ 2020 [1]. Eight months later, COVID-19 has spread to more than 217 countries/areas/or territories, affected over 105 million people and resulted in at least two million deaths (Figure 1), with substantially growing numbers of infections and deaths as the pandemic progresses in the low- and middle-income countries (LMICs). Although 80% of all patients with confirmed COVID-19 may have only mild to moderate symptoms, the case-fatality ratio is highly variable depending on how the cases are defined, the testing practices, the health system access and other unknown factors. Reports provide a range between 2–7% mortality overall and may be as high as 20% in the elderly [2]. Even within certain regions such as Africa the case-fatality rate due to COVID-19 ranged from 5.2% in Liberia to 8.6% in Chad [3].

The interaction between COVID-19 and the cardiovascular system has been a subject of special attention. The involvement of the cardiovascular system had already been described in other epidemics caused by corona viruses [6]. In COVID-19, patients with or at risk for cardiovascular disease (CVD) seem to be at greater risk of severe disease needing admission to an intensive care unit (ICU) and excess mortality [7,8]. Also, there have been reports of cardiac complications in COVID-19 patients including myocardial injury, acute coronary events and heart failure. It has been reported that patients with these complications (including troponin elevation) are at a higher risk of mortality [9].

**Figure 2:**
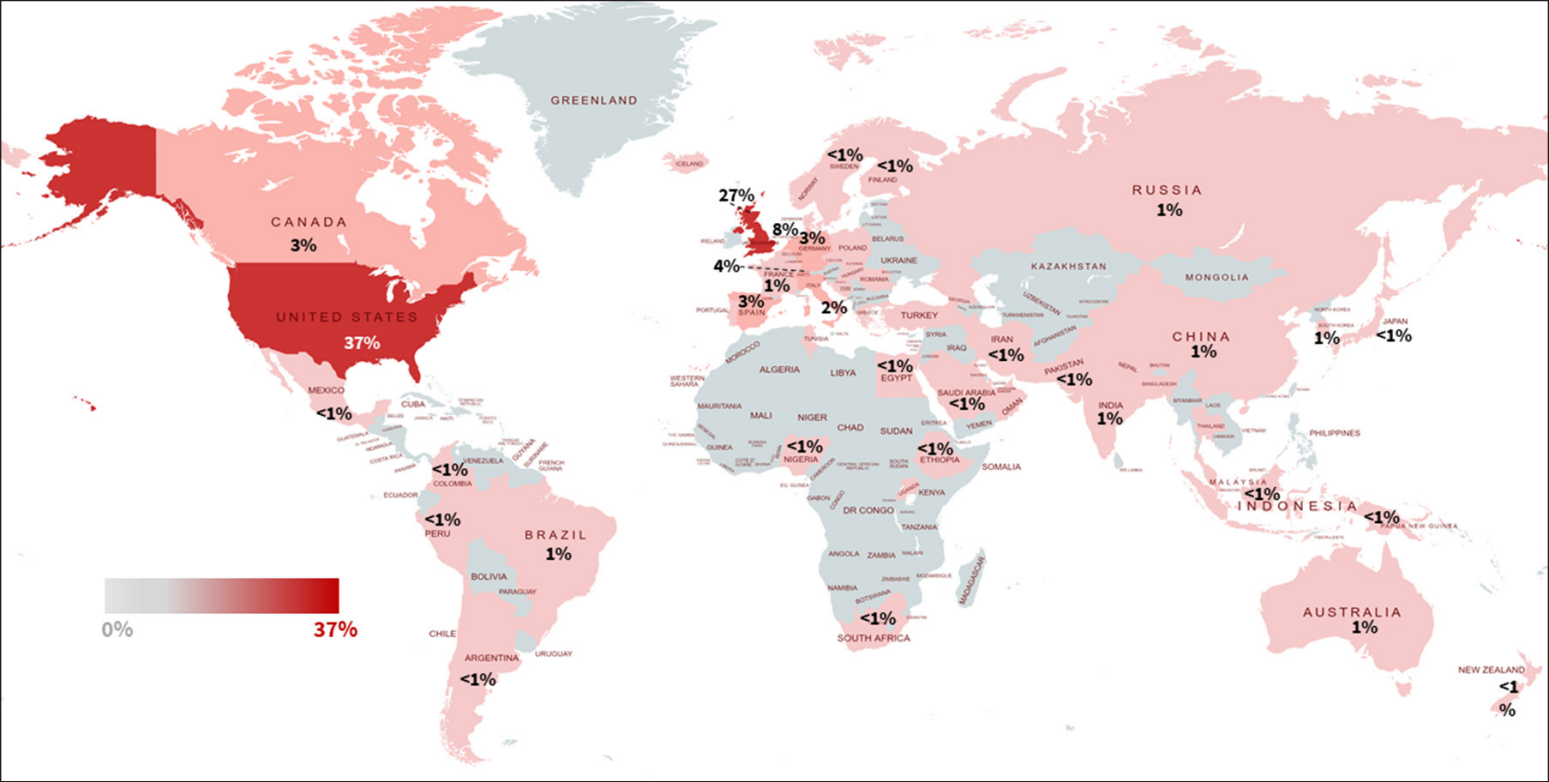
COVID-19 research output (in percentage) – Bibliometric analysis of the WHO COVID-19 research database.

Most of the published data about the epidemiology and management of patients with COVID-19 infections are published from high-income countries. Figure 2 highlights that nearly 95% of the published original research papers are from the United States, Europe and Western Pacific region (mostly from China and Japan) whereas limited data are reported from other low- and lower middle-income regions e.g., Africa only form 0.4%.

**Table:** 

Regions	N	Percentage

European Region	25,145	52.1
North America	19,271	39.9
Western Pacific Region	1,671	3.5
South-East Asia Region	882	1.8
South America	683	1.4
Eastern Mediterranean Region	432	0.9
African Region	187	0.4
**Total**	**48,271**	**100.0**

N = total number of original research publications. **Data source:** https://www.who.int/emergencies/diseases/novel-coronavirus-2019/global-research-on-novel-coronavirus-2019-ncov. * Search strategy run on 04 February 2021 with no restriction to language, publication year or journal/database. We only included original research studies related to risk factors, prognostic, diagnostic, aetiology, and observational studies, case report, screening study, prevalence, incidence, randomized trials, implementation research, qualitative research or health economic evaluations.

However, over 90% of the poorest billion live in LMICs, and about 80% of them are younger than 40 years as highlighted in the recent *The Lancet NCDI (non-communicable diseases and injuries) Poverty Commission* report [10,11]. Many CVDs such as rheumatic heart disease, congenital heart disease, peripartum and other cardiomyopathies, common contributors to CVD in LMICs lead to heart failure and premature death in young populations [12,13]. It is currently unknown if patients in LMICs and LICs regions with cardiovascular diseases are vulnerable to COVID-19 leading to poor outcomes.

The management of more seriously ill patients with COVID-19 and CVD requires resources, like medical supplies, ventilators, personal protective equipment (PPE), that could become limited, even in high-income countries. Understanding how countries where resources are scarce are dealing with the Pandemic is fundamental.

## Rationale for the current study

As summarized above although there is emerging evidence that CVD, diabetes, and hypertension is associated with COVID-19 and disease severity, SARS-CoV-2 itself may be cardiotropic in a subset of infected patients. Both acute and pre-existing CVD impact outcomes unfavourably. However, studies so far have, perforce, been conducted with important limitations (e.g. small numbers, limited geographical representation, lack of data standardization for risk factors and outcomes, limited measurement, lack of appropriate adjustment for important confounders, not including CVD conditions common in LMICs, and missing data).

In order to reach robust conclusions that could inform clinical and policy practices, we initiated a global study for a better understanding of the cardiovascular conditions that increase the risk of developing severe COVID-19, and a better characterization of cardiovascular complications in hospitalized patients with COVID-19.

### Study Objectives

#### Primary objectives

- To describe *cardiovascular outcomes* among patients hospitalized with COVID-19;
- To identify *cardiovascular risk factors* associated with poor in-hospital prognosis among patients with COVID-19.

### Methods

#### Study design

We will conduct a cohort study including consecutive confirmed COVID-19 patients in multiple countries.

**Study setting:** Participants will be recruited in any hospital where COVID19 patients are hospitalized. We have invited all WHF members from 100+ countries to identify three recruiting centres in their respective countries. Each centre should recruit between 50 and 500 consecutive patients depending on the size of the country.

**Study Population**

#### Eligibility criteria

##### Inclusion Criteria

All adult (as locally defined) with confirmed PCR positive COVID-19 infection who are hospitalized are eligible.

##### Exclusion criteria

Patients for whom we are unable to obtain informed consent will be excluded.

Patients who are unlikely to stay in the recruiting centre for 30 days (i.e. likely to be transferred) due to personal reasons or severe complications and unlikely to complete the 30-day follow-up visit.

#### Follow up

All patients will be followed up until 30 days, death or discharge whichever occurs first. If patient is discharged prior to 30 days, a phone contact will be made to find out whether patient is alive or dead (with cause) and if the patient had any re-hospitalization.

Table 1 reports the study measures included in this study and data collection source. In addition, each centre will provide the following information once at the beginning of the study: estimated size of population served, total number of beds, number of ICU beds, number of ventilators, number of cardiologists, availability of echocardiogram (including point-of-care ultrasound), number of primary PCI cases/ECMO/heart transplantations performed per year prior to the COVID-19 outbreak.

**Table 1:** Study measures and correlating data collection source for WHF Global study on COVID-19 and CVD.

Study Measure	Details	Data collection source

**PART 1: ENTRY FORM**		
**Demographics**	Age, sex, ethnicity, education, smoking status, pregnancy status	eCRF & hospital records
**COVID-19 symptom onset and admission vital signs**	Symptom onset, admission date, temperature, oxygen, respiratory rate, blood pressure, height, weight, waist circumference, shortness of breath	eCRF & hospital records
**Comorbidities (cardiovascular related prior to admission)**	Coronary artery disease, stroke, peripheral vascular disease, atrial fibrillation, heart failure, cardiomyopathies, rheumatic heart disease, chagas disease, valvular disease, hypertension, diabetes	eCRF & hospital records
**Comorbidities (non-cardiac related prior to admission)**	Chronic pulmonary disease, asthma, tuberculosis, HIV, renal replacement therapy, chronic kidney disease	eCRF & hospital records
**Cardiovascular medication (Pre-admission)**	Beta-blockers, alpha blockers, diuretics, ACE-inhibitors, anti-coagulants, anti-platelets, ARB, calcium antagonists, aldosterone antagonists, endocarditis prophylaxis, nitrates, statins	eCRF & hospital records
**Non-cardiovascular medication (Pre-admission)**	Anti-diabetic drugs, NSAIDS, allopurinol, anti-depressants, anti-retroviral therapy, influenza vaccine in the past 6 months	eCRF & hospital records
**Laboratory tests on admission**	Complete blood count, liver function tests, kidney function tests, cardiac biomarkers (Troponin-T, I, NT proBNP, CK-MB), lipids	eCRF & Lab reports
**Examinations**	ECG, ECHO, Chest-X-ray, CT-Scan Anonymized ECG data will be uploaded to a web-based platform to be read and codified in a centralized reading centre by experienced and certified cardiologists.	eCRF & hospital records
**Medications during hospitalization**	Intravenous fluids, antivirals, ACE-I, ARB, NSAIDS, antibiotics, anti-malarial agent, anti-fungal agent, corticosteroids	eCRF & hospital records
**Supportive care during hospitalization**	ICU admission, oxygen therapy, non-invasive and invasive ventilation, inotropes, extracorporeal support	eCRF & hospital records
**PART 2: OUTCOME FORM**		
At discharge	Blood pressure, heart rate, time in ICU, cardiovascular events, acute respiratory distress syndrome, pneumonia, acute renal injury, liver dysfunction, death (cause of death)	eCRF & discharge summary
30-day follow-up	Vital stats (alive or death), re-hospitalization, recovery from COVID-19	Phone or clinic visit
**Hospital and Provider level information**	Hospital facilities (hospital beds, ICU, ventilators), availability of services (ECG, ECHO, x-ray, CT-scan, cardiac cath lab) human resources (cardiologists, pulmonologists, infectious disease specialists)	Hospital records

ACEi = angiotensin-converting enzyme inhibitor, ARB = angiotensin II receptor blocker, ECG = electrocardiogram, ICU = intensive care unit, NSAIDS = non-steroidal anti-inflammatory drugs.

#### Outcomes

The outcomes that we propose to measure include the need of ICU admission, need of ventilator, death (with cause), major adverse cardiovascular events (myocarditis, arrhythmia, heart failure [including LVEF], acute coronary event [type of MI]; neurological outcomes, acute renal failure, and pulmonary outcomes).

#### Data collection

This study will be coordinated from the Public Health Foundation of India (PHFI) and Centre for Chronic Disease Control (CCDC) (India) and conducted in hospitals in low-, middle- and high-income countries. Data will be collected at each site by local investigators and sent to the coordinating centre. Only data outlined on the entry (CRF Part 1) and outcome forms (CRF Part 2) will be collected. Each site will have a research coordinator who will enter in-hospital entry and outcome form via a secure website throughout the entire study period. Hospital-level data will be collected just once when the hospital joins the study.

Study data will be collected and managed using the electronic data capture platform REDCap hosted at PHFI [14,15]. REDCap is a secure, web-based software platform designed to support data capture for our study by providing 1) an intuitive interface for validated data capture; 2) audit trails for tracking data manipulation and export procedures; 3) automated export procedures for seamless data downloads to common statistical packages; 4) procedures for data integration and interoperability with external sources; and 5) safe and secure storage of all the audio files and study-related field-notes. The entry form will be used at hospital admission to collect baseline data. The outcome form will be completed 30 days after hospital admission or death or hospital discharge whichever occurs first. These data will be collected from the patient’s routine medical records and no special tests will be required. We will also collect data on ECG (scanned copies of ECG and/or digital files) and echocardiogram (raw DICOM) conducted as part of the usual clinical care of patients. ECG exams will be uploaded to a web-based platform to be read and codified in a centralized reading centre, [16] according to the Minnesota Code, by experienced and certified cardiologists. Automatic measurements of ECG intervals, including the QT interval, will be reviewed. ECG (xml or image files) and echo images (raw DICOM) will be anonymized at sites via provided software and sent via encrypted cloud for central reading. All vetted researchers will be given free access to the online platform and machine learning tools to do further research.

### Data management

#### Source Data

Data will be collected directly from the patient’s routine medical records, clinical notes at hospital admission, and at 30 days, discharge or death, whichever occurs first, using CRFs administered by study investigators. If a patient is discharged before 30 days investigators will make a phone contact to find out whether patient is alive or death at 30 days and if the patient had re-hospitalization. The CRF (source notes) will be completed in English for each enrolled patient. Study personnel and the principal investigator at each centre will be responsible for evaluating the CRFs for accuracy and completeness before entering into the online electronic clinical data management system. Once entered, the data manager will review the data for discrepancies and missing data. The site will then be informed to make any required corrections and/or additions and the CRF’s stored until all analyses are completed. The principal investigators and the study site research coordinator will be required to respond to the data queries and confirm and correct the data. A printed copy of the CRF and individual informed consents will be maintained in the participant’s file. Confidentiality of participant’s data will be maintained in accordance with national laws with the informed consent form containing a statement describing the extent to which confidentiality of the participant will be maintained.

#### Database

Study personnel at the participating sites will be responsible for completing CRFs through remotely accessing a centralized electronic clinical data management system (Redcap). Appropriate training will be provided to the clinic site teams for entering data into the electronic data capture system by the project manager. PHFI/CCDC will be serving as the Research Coordinating Centre and will have access to all the study data. Individual sites will have access to their own centres’ data and will be provided site-specific data summaries by CCDC’s data management team, upon request.

#### Access to data, ownership, disclosure of data and publication

The Sponsor and coordinating centre (PHFI/CCDC) are responsible for storage, protection and retrieval of registry data. The Steering Committee is responsible for the guardianship and use of the data. PHFI/CCDC will have access to all the study data. Individual sites will have access to their own centres’ data. Each site personnel who is responsible for entering and reviewing participant data on the Electronic Clinical Data Management System will be provided access with a secure password. Sites will be provided with site-specific data summaries by PHFI/CCDC, upon request. All publications will be approved by the Steering Committee who will be named on all reports. The research teams, research nurses, collaborating doctors, and their respective units will be named, and study participants acknowledged in the final report and in publications arising from this registry. Acknowledgement in a future publication will be offered to all participating centres, national leaders, and site investigators that have provided valid data sets, according to the World Heart Federation Steering Committee.

#### Sample size and analysis

We will invite all WHF members (Scientific Societies and Foundations) from 100+ countries to take part in this study. Assuming that 35 members identified at least two hospital sites, recruiting an average of 75 patients each, we will be able to recruit 5,200 participants. With this sample size, and assuming a prevalence of 8% for potential risk factor (hypertension), we will have the power to detect a RR of 2.0 for poor outcomes (myocarditis, and other major adverse cardiovascular events. We will also be able to estimate outcomes with 95% precision (confidence interval). A sample size of 5000 participants produces a two-sided 95% confidence interval with a width equal to 0.008 when the sample proportion (prevalence of cardiovascular risk factors) is 0.08.

**Statistical analyses:** Data will be reported as a number (proportion) of patients for categorical variables, mean (SD) for normally distributed continuous variables, and median (IQR) for skewed distributions. Data-derived and previously published multivariate models for in-hospital or post-discharge mortality and complications will be used to adjust for significant covariates.

#### Institutional Ethics Committee Approval

Institutional ethics approval for the project has been obtained from the University of Cape Town, South Africa and the coordinating centres in India (PHFI and CCDC). In addition, all investigators will obtain ethical approval from their institutional ethics committees prior to patient recruitment in the study. Eligible patients will be approached regarding potential participation in the study and the study purpose, risks, and potential benefits will be explained by the study coordinator and/or principal investigators. Potential patients will be given the opportunity to ask questions. Patients who voluntarily agree to participate in the study will be asked to document their informed consent.

#### Informed Consent

The investigator or the person designated by the investigator should fully inform the patient of all the important aspects of this observational study, including the approval by the ethics committee of the study protocol. Informed consent should be guided by local ethical requirement and in some institutions can be verbal. Prior to the participation of the patient in the study, the informed consent form should be signed and personally dated by either the patient or the patient’s legally acceptable representative, and by the person who conducted the informed consent interview. For those patients who are directly unable to provide written informed consent (due to admitted in ICU or unconscious), consent could be obtained from a legally acceptable representative and/or in the presence of an impartial witness if acceptable by local regulation. Informed consent process will be guided by the local ethics and regulatory requirements as applicable for the COVID-19 research projects.

#### Participant’s confidentiality

Study data sent to the Electronic Data Management System at the Research Coordinating Centre at CCDC will be securely stored. Source documents pertaining to the study which is maintained in each participating site will be stored securely by the site coordinator, in accordance with the ethical requirements. Informed consent will be obtained from the participants to collect, transmit and store identifiable personal information.

Identifiable data collected during this study will be replaced by a code number. This number will be used for study documentation, in reports or publications. The study data once analysed and published will be stored for a maximum of 15 years.

#### Study Management and governance

##### Principal Investigators

Dorairaj Prabhakaran, Karen Sliwa, Pablo Perel.

##### Co-investigators

Fausto Pinto, Dike Ojjii, Carolyn Lam, Friedrich Thienemann, Junbo Ge, Amitava Banerjee, Kristin Newby, Antonio Luiz Ribeiro, Samuel Gidding.

**National Leaders and site investigators** will constitute the larger team contributing to this cohort study and will be acknowledged to future publications as per the ESC and EOPR publications and authorship policy.

#### Study Steering committee

The **Steering Committee** will be the decision-making body of this registry. It will consist of members of PHFI and CCDC, India (DP, KS), LSHTM, UK (PP) and University of Cape Town, South Africa (KS) will meet, via telephone conference, on approximately a weekly basis (or less frequently if deemed appropriate) to address the operational and scientific matters of the study. The Steering Committee will make the major decisions regarding the project, including: the scientific direction of the project, including partner responsibilities and any changes in budgetary allocations, the dissemination and exploitation strategy; and any administrative and legal decisions relating to the registry’s contractual obligations to the study sponsor (World Heart Federation).

The daily administrative, scientific and technical management of the project will be executed by a **Project Management Team** based at PHFI/CCDC, consisting of the Principal Investigator (DP), co-investigator (KS) and the Project Manager. The Project team will have support by the World Heart Federation. The Project Management Team will help carry out the decisions of the Steering Committee and will be supported in its tasks by the administrative resources based at PHFI/CCDC.

#### Study implications

This global cohort study will provide insights into the cardiovascular outcomes and cardiovascular risk factors among hospitalized patients with confirmed COVID-19. In addition, this study will highlight the ethnic difference and health system differences in poor cardiovascular outcomes in hospitalized patients with COVID19. By providing comparable data from countries around the globe, the study will inform the delivery of care for patients with COVID-19, with underlying cardiovascular conditions or with cardiovascular complications as well as will contribute to potential meta-analysis with data from other global registries focused on COVID-19 and CVD outcomes. This study has additional implications. Recent data from UK shows ethnic differences in the need for hospitalization and differences in the incidence of death. For example, a study showed black patients had a higher odds of hospitalisation but similar death rates compared to white patients while South Asians had higher odds of death once hospitalized but similar rates of hospitalisation as compared to whites [17]. These findings were observed in an advanced health system. Our study has the potential to compare both ethnic differences as well as health system variations and also contribute to meta-analysis from multiple studies.

## Participating site investigators and research teams who entered the first 500 patients

**Buenos Aires, Argentina**, Hospital de Clinicas of the University: R. Pérez de la Hoz, J. Martin Aladio, M. Matsudo, S. Swieszkowski, A. Perez de la Hoz, Costa, **Buenos Aires, Argentina**, Sanatorio Güemes: E. J. Zaidel, J. Perea, M. Ariel Oliva, N. Carboni Martinez, N. Bisso, L. Gheco, **Srpska, Bosnia and Herzegovina**, University Clinical Center Republic of Srpska: B. Stanetic, I. Ovcina, B. Dujakovic, R. Tamburic, **Colombia**, Clinica de Occidente, J. Lugo-Peña, M. Ángel Zuleta, **Colombia**, ISSSTE Clínica Hospital de Guanajuato, J. E. Luna Cárdenas, G. Sánchez Loza **Kumasi, Ghana**, Komfo Anokye Teaching Hospital: L Tetteh Appia, Y. Hardy, J. Hutton, **Accra, Ghana**, Military Hospital: A. Toppar, **Ludhiana, India**, Dayanand Medical College Hospital: B. Mohan, M. Mennen, S. Singla, K. Jain, Ankush, **Hyderabad, India**, Apollo Hospital: V. Ram, G. Praveen Kumar, K. Subba Reddy, B. V. K. S. Sowmya, M. Rebecca **Isfahan, Iran**, Amin Hospital: N. Sarrafzadegan, L. Sadeghian, Khorshid hospital, **Iran**, S. Mohammad Hashemi, S. Hashemi, **Kyoto, Japan**, Kyoto Medical Center: K. Hasegawa, Y. Iida, **Mombasa, Kenya**, Coast General Teaching and Referral Hospital: E. Ogola, K. Mwazo, V. Vaghela, S. Mohamed, A. Abeid, V. Mumbo, M. Ali Mohamed, A. Ikbal Varvani, M. Omar, V. Karegi, B. Nduati, Swaleh, E. Gacheri, D. Anyanga, S. M. Mohamed, E. Gacheri Riungu, D. Anyanga, **Ibadan, Nigeria**, University College Hospital: O. Ogah, O. Adekanmbi, O. Adebayo, Y. Oyebisi, O. Makinde, O. A. Orimolade, O. Makinde, S. Alabi, **Sagamu, Nigeria**, Olabisi Onabanjo University Teaching Hospital: F. Inofomoh, **Sindh, Pakistan**, Tabba Heart Institute: S. Sheikh, R. Malik, Z. Khan, S. Fateh, **Lisbon, Portugal**, University Hospital Sta Maria: F. Pinto, D. Caldeira, S. Braz, J. Agostinho, J. Brito, H. Barbacena, F. Parlato, C. Carreiro, R. Soares, C. Gomes, A. Pinto Sousa, M. José Pires, **Cape Town, South Africa**, Groote Schuur Hospital: N. Ntusi, L. Chinhoyi, O. Briton, C. Viljoen, K. Sliwa, P. Singh, L. Chinhoyi, M. Mennen, Khartoum, Sudan, Fedail Hospital: A. Suliman, **Lusaka, Zambia**, Levy Mwanawasa University Hospital: F. Goma, N. Mumba.

Research Coordinating Centre: PHFI/CCDC, India.

Dimple Kondal (Senior Biostatistician), Mumtaj Ali (Data Manager), Snehalata Roy (Research Assistant).
